# Sulfur Molecules in Space by X-rays: A Computational Study

**DOI:** 10.1021/acsearthspacechem.0c00238

**Published:** 2021-02-24

**Authors:** Goranka Bilalbegović, Aleksandar Maksimović, Lynne A. Valencic, Susi Lehtola

**Affiliations:** † Department of Physics, Faculty of Science, University of Zagreb, Bijenička cesta 32, 10000 Zagreb, Croatia; ‡ Center of Excellence for Advanced Materials and Sensing Devices, Rudjer Bošković Institute, Bijenička cesta 54, 10000 Zagreb, Croatia; § NASA Goddard Space Flight Center, Greenbelt, 20771 Maryland, United States; ∥ Department of Physics & Astronomy, The Johns Hopkins University, 366 Bloomberg Center, 3400 N. Charles Street, Baltimore, 21218 Maryland, United States; ⊥ Department of Chemistry, University of Helsinki, P.O. Box 55, A. I. Virtasen aukio 1, FI-00014 Helsinki, Finland

**Keywords:** molecules in space, astrochemistry, X-ray spectra, interstellar medium, algebraic-diagrammatic construction, density functional theory, coupled cluster methods

## Abstract

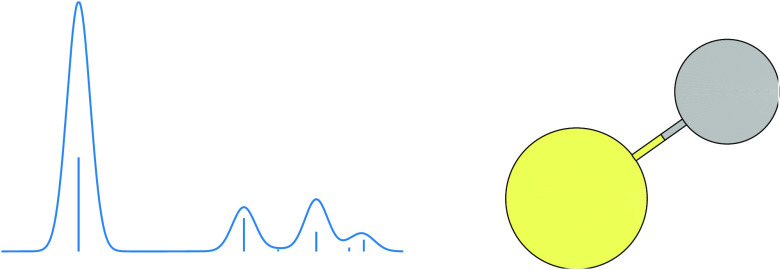

X-ray astronomy lacks high resolution spectra of interstellar dust analogues and molecules, severely hampering interstellar medium studies based on upcoming X-ray missions. Various theoretical approaches may be used to address this problem, but they must first be shown to reproduce reliable spectra compared to the experiment. In this work, we calculate the sulfur K edge X-ray absorption spectra of H_2_S, SO_2_, and OCS, whose spectra are already known from X-ray experiments and predict the X-ray spectrum of CS, which as far as we are aware has not been measured, thereby hampering its detection by X-ray telescopes. We chose these four molecules as the astrochemistry of sulfur is an unsolved problem and as the four molecules are already known to exist in space. We consider three types of methods for modeling the X-ray spectra: more accurate calculations with the algebraic-diagrammatic construction (ADC) and the CC2, CCSD, and CC3 coupled cluster (CC) approaches as well as more affordable ones with transition potential density functional theory (TP-DFT). A comparison of our computational results to previously reported experimental spectra shows that the core–valence separation (CVS) approaches CVS-ADC(2)-x and CVS-CC3 generally yield a good qualitative level of agreement with the experiment, suggesting that they can be used for interpreting measured spectra, while the TP-DFT method is not reliable for these molecules. However, quantitative agreement with the experiment is still outside the reach of the computational methods studied in this work.

## 1 Introduction

X-ray absorption experiments have long been used to identify chemical species in materials science, even in situations where the absorption features belong to the same range of energies.^1^ As a result, X-ray studies are quite developed for complex materials of interest in chemistry, physics, and biology.

X-ray studies are useful also in extraterrestrial contexts: observations with X-ray telescopes such as Chandra and XMM-Newton have already substantially improved our knowledge of various astrophysical phenomena.

Although the detection of molecules in space is traditionally based on the use of radio and infrared telescopes,^2,3^ the use of the X-ray range provides information on the molecule or grain composition which is not available from the traditional long wavelength regimes.

However, the interpretation of the observed X-ray absorption near edge structure (XANES) spectra is critically dependent on the quality of the reference spectra used for the identification, and the procedure thus hinges on the availability of high-quality reference spectra for the molecules under study in relevant environments.

Reference spectra for astrophysical observations typically come from laboratory measurements of dust and molecules. However, laboratory astrophysics for dust and molecules in space is less developed for X-ray spectroscopy than for other wavelengths: only some measurements of dust materials have been performed with modern synchrotron sources.^4−9^ In addition, significant contributions to the measured astrophysical spectra may arise from radicals as well as charged species, which tend to be challenging to study experimentally.

As an alternative, calculated spectra can also be used for identification:^10,11^ computational studies are straightforward even for species whose measurement is difficult; however, in order to use computed spectra, one must first establish the accuracy of the computational model by comparison to known spectra.

To facilitate future observations in X-rays and aid possible detection of new species, in this work, we study X-ray absorption spectra at the sulfur K edge of H_2_S, SO_2_, OCS, and CS. We have chosen these four molecules for several reasons. The molecules are small, which allows the use of sophisticated computational methods to model their X-ray spectra. Experimental spectra have been reported for H_2_S, SO_2_, and OCS, allowing us to study the reliability of our computational models, while for CS, we make a prediction, as to the best of our knowledge, the spectrum of CS has not yet been measured. The four molecules are also already known to exist in space. CS was the first molecule discovered in space.^12^ Shortly thereafter, OCS was detected in the Sgr B molecular cloud,^13^ and interstellar H_2_S was discovered along the line of sight of seven galactic sources.^14^ SO_2_ was detected for the first time in observations along the line of sight of Orion and Sgr B2.^15^ The four molecules have been later observed in many other astrophysical environments, as well, including the Solar system, our Galaxy, and external galaxies.^2,3^

We have also chosen these molecules because very little is presently known about the astrochemistry of sulfur. Sulfur is known to be depleted from the gas phase in dense molecular clouds. It could exist in the solid phase, but only small amounts of H_2_S, SO_2_, and OCS have been observed in dust grains.^16−18^ Sulfur could also hide in metal compounds, such as FeS.^19^ Kama et al.^20^ recently studied 16 young, disk-hosting stars and found that (89 ± 8)% of sulfur in the inner regions of disks is in the form FeS and other sulfide minerals. Some sulfur does exist in the gas phase either in neutral atomic S or cationic S^+^ forms and in small molecules.^2,3,21−25^ There is also a possibility that undiscovered larger sulfur molecules exist in the gas phase^26^ or in dust grains.^27,28^

Having laid out our goals for this work, we would like to shortly discuss the state-of-the-art X-ray spectrum calculations. Several theoretical methods for calculating X-ray spectra are available in the literature, such as many-body perturbation theory based on the Bethe–Salpeter equation,^29,30^ coupled cluster (CC) theory,^31−36^ restricted open-shell density functional theory (DFT),^37^ orthogonality constrained DFT,^38^ nonorthogonal configuration interaction calculations,^39^ time-dependent DFT,^40,41^ algebraic-diagrammatic construction (ADC),^42−46^ and transition potential (TP) approximation^47^ of DFT.^48,49^ Each of these methods produces good agreement with experiments for some systems, whereas for other systems, the methods’ errors may be larger. Even though the errors in, for example, CC calculations and the ADC can be systematically reduced by decreasing the amount of truncations involved in the model, this results in a significant increase of the required computational effort, limiting the more accurate calculations to few-atom molecules.

Many of these methods are in any case computationally demanding and are thereby untractable for routine modeling of many molecules found in space, such as the C_60_ and C_70_ fullerenes.^50^ Cosmic dust grains also have astrophysical significance and can be modeled either as nanoparticles or as crystals; in either case, such calculations require explicit modeling of many atoms and are thus computationally challenging. Once new telescopes are launched, it is likely that several more large molecules in the gas phase or dust form will also join the list of known molecules in space, requiring computationally tractable approaches for large systems.

In this work, X-ray spectra are calculated with TP density functional theory (TP-DFT) as well as with the ADC and CC methods. TP-DFT builds on DFT, which is the main workhorse of present-day materials science and quantum chemistry due to its good accuracy in calculating the properties of systems of interest in various branches of science and technology.^51,52^ As TP-DFT is routinely applicable to extended systems, it is considered to be suitable as a general-use tool for computations of X-ray spectra for astrophysical molecules and dust grains. In contrast, the ADC^42−46^ and CC^31−33,35^ schemes, which offer a systematical hierarchy of methods for approaching the exact solution to the Schrödinger equation, are considerably more expensive. However, CC and ADC often yield spectral intensities and spectral shapes that are in better agreement with the experiment than those from TP-DFT.

Next, in Section 2, we will discuss the computational procedures to obtain the spectra of H_2_S, SO_2_, OCS, and CS with the TP-DFT, ADC, and CC approaches, which are presented and compared with available X-ray experiments in Section 3. The article ends with a discussion in Section 4 and conclusions in Section 5.

## 2 Computational Methods

The molecular structures of H_2_S, SO_2_, OCS, and CS were optimized at the ωB97M-V/aug-pcseg-2 level of theory^53−56^ with a development version of the Q-Chem package,^57^ version 5.2, employing default settings; the optimal geometries are available in the Supporting Information. All experimental spectra used in this work were digitized from the original publications by WebPlotDigitizer version 4.3^58^ employing the “X step with interpolation” algorithm.

The ADC, TP-DFT, and CC calculations produce a stick spectrum for the transitions between different states; the stick spectra for the ADC and CC calculations are reported in the Supporting Information. To model the available experimental spectra of H_2_S, SO_2_, and OCS for life-time and experimental resolution effects, the computed stick spectra with intensities *I*_*i*_ at energies ω_*i*_ are convoluted by a Gaussian function
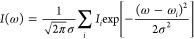
1with a broadening factor of σ = 0.3 eV for H_2_S and SO_2_ and CS; these broadenings were determined to yield good agreement with the measured spectra. As we are not aware of an experimental spectrum for CS, we use the same broadening factor σ = 0.3 eV also for this molecule, in order to aid comparisons to future measurements. Spectra for a much smaller Gaussian broadening of σ = 0.1 eV, as well as spectra with Lorentzian broadening, are presented for all four molecules in the Supporting Information. In this work, broadened spectra are shown in combination with the stick spectra, in which the intensities for degenerate transitions are shown summed together.

To ease visual comparison with experiment, the computed, broadened spectra are shifted so that the first maximum is at the same position as in the experiment; the value of the used energy shift is given in each figure caption. Because we are not aware of an experimental spectrum for CS, the computed spectra for CS are shown relative to the corresponding sulfur K edges.

Because X-ray spectra are traditionally reported in arbitrary units, for simplicity, the experiment and computational predictions are mapped to the same scale by normalizing the maximum intensity of the first peak to unity in both the experimental spectrum and the broadened theoretical spectra.

### 2.1 ADC Method

The ADC approach to electronic excitations is based on the perturbation expansion of the polarization propagator.^42,45^ ADC methods are perturbation schemes that are in principle improvable whenever the perturbation series converges; the ADC(*n*) scheme corresponds to the *n*-th order of perturbation theory. The ADC equations are derived either using the many-body Green’s functions theory^42^ or in an intermediate-state representation.^59^ The ADC calculations were carried out with the ADC-connect (ADCC) program, version 0.15.1,^60^ in combination with PySCF, version 1.7.1.^61^ Scalar relativistic effects were described with the exact two-component (X2C) method.^62^ A point nuclear model was used in the calculations, as differences to the Gaussian nuclear model were found to be negligible for the molecules studied herein. Testing and comparing a number of ADC approximations led us to adopt the ADC(2)-x approach, which is known to produce X-ray spectra in good agreement with experiments.^44,46^

The core–valence separation (CVS) approximation is employed, as usual, in order to make the calculations tractable in the X-ray regime. The CVS approximation neglects the interaction between core and valence excitations, which are typically small due to the large separation in energy between the core and valence orbitals. The accuracy of the CVS approximation was also investigated, as a recent study showed that errors of the CVS approximation in the ADC methods for various basis sets range from −0.4 to +0.7 eV for elements in the second and third periods.^63^ However, following the methodology of ref (63), we found the CVS error around the sulfur K edge to be negligible (∼0.01 eV) for the higher-level, fully decontracted basis sets used in this work, justifying the use of the CVS approximation.

A detailed basis set convergence study was performed for the CVS-ADC(2)-x spectra. Decontraction and augmentation of the basis set were found to be important, and the decontracted augmented quadruple-ζ aug-pc-3 basis set^53−55^ (denoted: un-aug-pc-3) was found to yield sufficiently converged spectra; similarly, converged results were also obtained with the decontracted augmented quadruple-ζ aug-cc-pV(Q+d)Z basis set^64−66^ (un-aug-cc-pV(Q+d)Z; see Supporting Information).

The convergence of the CVS-ADC(2)-x spectra with respect to the number of calculated states was also studied. We chose to include 10 excited states for the calculations on H_2_S and CS and 20 excited states for the calculations on SO_2_ and OCS.

Additional CVS-ADC(2)-x calculations were carried out for H_2_S and CS using doubly and triply augmented basis sets^67^ (un-daug-pc-3 and un-taug-pc-3), in order to assess the importance of Rydberg transitions on the spectrum. Although it is traditionally difficult to use multiply augmented basis sets in molecular calculations due to issues with numerical instabilities caused by the resulting overcomplete basis set,^68^ this issue has been recently resolved via a procedure based on pivoted Cholesky decomposition^68,69^ that has been implemented in PySCF. We found that the importance of Rydberg states on the X-ray spectra of the studied molecules is small; the spectra for the un-daug-pc-3 and un-taug-pc-3 calculations are presented in the Supporting Information.

Comparison of the stick spectra in the un-aug-pc-3 and its multiply augmented counterparts reveals that multiple augmentation has negligible effect on the energies and intensities of the first 6 transitions in both H_2_S and CS. Notable differences in the spectra only arise later on, where the spectrum is characterized with a large number of tightly spaced transitions with weak intensities, especially in the calculations with multiply augmented sets. This region corresponds to transitions into the continuum, where calculations in Gaussian basis sets are not expected to be reliable. This conclusion is also supported by the analogous CC calculations on all four molecules; see Section 2.3.

### 2.2 TP Density Functional Theory

X-ray absorption spectra were also calculated using TP-DFT with the ERKALE software package (git snapshot 6ed6aa1).^70^ TP-DFT relies on the choice of density functional approximation, which describes the quantum mechanical interaction of the electrons. Hundreds of density functional approximations have been published in the literature,^71^ but the reliability of the resulting computational predictions is system dependent. The role of the exchange–correlation functional in TP-DFT calculations of X-ray spectra has been studied previously for various chemical species.^72,73^ We assessed several functionals and basis sets in calculations of K edges of H_2_S, SO_2_, and OCS to determine the optimal approach. The entire hierarchical family of the polarization consistent basis sets of Jensen^53−55^ was benchmarked for the accuracy of the position of the first peak and the overall shape of the spectrum with several exchange–correlation functionals. It was again found that fully decontracting the basis set on the excited sulfur atom led to the fastest convergence and that the decontracted augmented triple-ζ aug-pc-2 basis yielded sufficiently converged results (peak position converged to ∼0.1 eV); however, un-aug-pc-3 is used for a consistent comparison to the CVS-ADC and CVS-CC results. The double-basis set procedure in which a large set of Rydberg primitives is placed on the excited atom was also used in the TP-DFT calculations to improve the description of unoccupied continuum states.^74,75^ We selected the ωB97M-V range-separated functional^56^ for the spectra shown in this work, as we found this functional to reproduce the best agreement with experiment. The ERKALE calculations employed a (99,590) integration grid for the local exchange–correlation contributions and a (50,194) grid for nonlocal correlation contributions.

The absorption spectra are obtained from the TP approximation,^47^ which produces estimates for all transitions from a single calculation. The main drawback of TP is that it does not produce a reliable absolute energy scale, as it does not fully model the relaxation of the core hole. However, an absolute energy scale can be established with an explicit calculation of the first core-excited state with a ΔSCF [Δ self-consistent field] procedure, in which the 1s electron is moved onto the lowest unoccupied orbital; ΔSCF calculations are well-known to afford rather accurate estimates for excitation energies. Next, the computed TP spectrum is shifted so that the first transition occurs at the energy obtained from the ΔSCF calculation.^76^ Finally, the calculated stick spectra are again broadened with Gaussian functions, as discussed above in Section 2.

As an element of the third period, the 1s orbital of sulfur experiences a considerable relativistic effect. Although this effect could be captured, in principle, with, for example, the X2C method used in the CVS-ADC calculations, we are not aware of any programs that support TP-DFT calculations with this approach. Instead, previous studies of X-ray absorption spectra of sulfur molecules within TP-DFT^77−79^ applied a relativistic correction of +7.4 eV obtained by Risberg and co-workers.^78^ Repeating the ωB97M-V/un-aug-pc-3 ground-state calculations within PySCF, both with and without the X2C correction, we find that the scalar relativistic correction to the sulfur 1s orbital energy is 7.3 eV for all molecules, as shown by Table 1. Analogous to the previous works mentioned above, relativistic effects were modeled by shifting the nonrelativistic spectrum from ERKALE by +7.3 eV.

**Table 1:** Relativistic Shifts in the Ground State Sulfur 1s Energy Calculated for the ωB97M-V Functional and the Decontracted aug-pc-3 Basis

molecule	correction (eV)
H_2_S	7.275
SO_2_	7.275
OCS	7.272
CS	7.272

### 2.3 CC Method

Similar to the ADC scheme, the CC method is a systematically improvable approach for solving the Schrödinger equation. Methods based on CC theory are becoming standard tools in applications of electronic structure theory for problems in chemistry and physics.^80,81^ Recent works using the CC method have the reproduced X-ray absorption spectra in good agreement with experiments.^31−35^

We use the e^T^ package, version 1.0.7, to compute X-ray spectra.^34,82^ The equation-of-motion method with the CVS technique is used with three coupled-cluster approximations: CC2 (CC singles and perturbative doubles),^83^ CCSD (CC singles and doubles),^84^ and CC3 (CC singles and doubles and perturbative triples).^34,85^ CC2 is an approximation to CCSD, while CC3 is an approximation to CCSDT (CC singles, doubles, and triples); the accuracy of the methods can thereby be formally classified as CC2 (least accurate) < CCSD < CC3 (most accurate). While the CC methods become more accurate in increasing rank, with systems with *n* electrons being described exactly with CC theory that includes up to *n*-fold substitutions, the computational cost also undergoes a steep increase at every step of the level.

We studied the basis set convergence of the CVS-CC2, CVS-CCSD, and CVS-CC3 spectra with the un-aug-pc-*n* basis sets (*n* = 0, 1, 2, and 3); the values of the corresponding K edges are given in the Supporting Information. The difference between the un-aug-pc-2 and un-aug-pc-3 K edges is of the order of 0.2 eV for all molecules, suggesting that the un-aug-pc-3 spectrum is sufficiently converged. Spectra for the un-aug-pc-3 basis are therefore used in this work. The CC3 calculations for SO_2_ failed to converge due to degenerate eigenstates. Therefore, for SO_2_, we only show spectra for the CC2 and CCSD methods, while for H_2_S, OCS, CS, and also CC3 data are included.

The convergence of the CC spectra with respect to the number of excited states was examined. Five excited states were sufficient for a converged spectrum for SO_2_; six states were necessary for CS and H_2_S, while seven excited states were used for OCS. The CC stick spectra are broadened with the approach given in Section 2.

Because relativistic corrections are not included in e^T^ at the moment, we employ a semiempirical shift obtained at the X2C level^62^ for Hartree–Fock using the Psi4 program,^86^ version 1.3.2. The resulting orbital energy shifts for the sulfur 1s are shown in Table 2. Based on these data, the CC excitation energies are shifted by + 7.9 eV to account for relativistic effects.

**Table 2:** Relativistic Shifts in the Ground State Sulfur 1s Energy Calculated at the Hartree–Fock Level and Using the Decontracted aug-pc-3 Basis

molecule	correction (eV)
H_2_S	7.915
SO_2_	7.917
OCS	7.911
CS	7.912

e^T^ employs a pivoted Cholesky decomposition of the atomic-orbital basis analogously to refs (68) and (69), allowing the use of overcomplete basis sets. As the basis set convergence of the CC methods is known to be similar, the importance of further augmentation of the basis, that is, the effect of Rydberg states was studied with the CVS-CC2 method. In agreement with the CVS-ADC calculations described in Section 2.1, multiple augmentation was found to have negligible effects on the CVS-CC spectra with the chosen number of excited states. The CVS-CC2/un-aug-pc-3 and CVS-CC2/un-daug-pc-3 stick spectra are given in the Supporting Information.

## 3 Results

### 3.1 H_2_S

The X-ray absorption spectrum of H_2_S has been measured in two works. Bodeur and Esteva^88^ found several peaks of decreasing intensity with increasing photon energy. The near-edge region was measured with an improved energy resolution by Reynaud et al.,^87^ with an overall shift of the spectrum of 0.4 eV in comparison to the previous measurement of ref (88). The experimental spectrum by Reynaud et al.^87^ has two major peaks around 2472.7 and 2475.7 eV.

**Figure 1:**
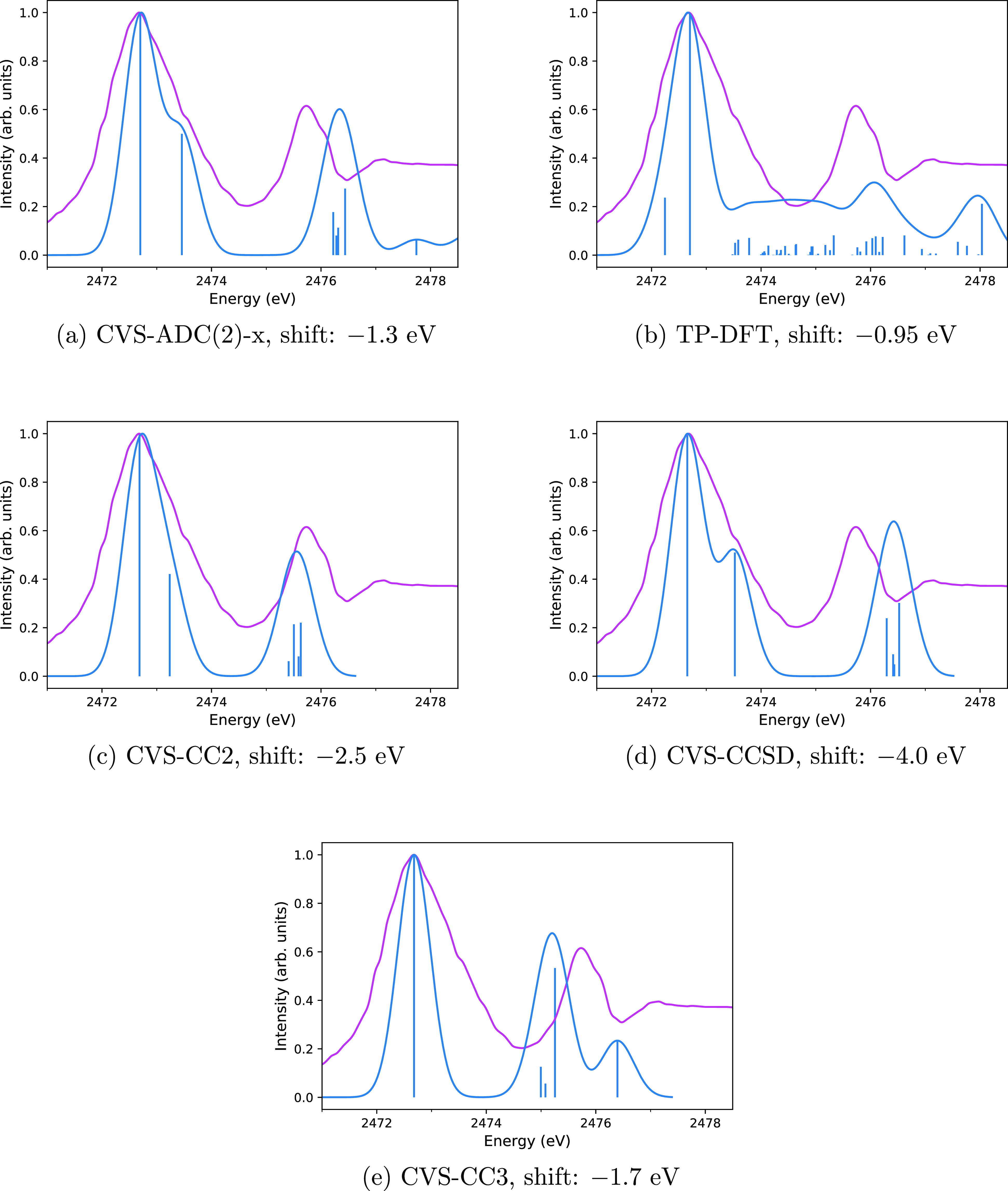
Comparison of computed (blue) and experimental (magenta) spectra of H_2_S with the CVS-ADC(2)-x (a), TP-DFT (b), CVS-CC2 (c), CVS-CCSD (d), and CVS-CC3 (e) methods. Computed spectra are translated by the amount shown in the captions to align the main peak to the experiment. Vertical lines represent the calculated spectra, whereas the curves are the theoretical spectra broadened for lifetime and detector resolution effects to mimic the shape of the experimental spectrum closely as possible. The experimental spectrum is adapted with permission from Reynaud et al.^87^ (copyright 1996 published by Elsevier B.V.).

The CVS-ADC(2)-x spectrum, which has been translated to match experiment as described in Section 2, is shown in Figure 1a. The CVS-ADC(2)-x spectrum agrees well with experiment. The first two CVS-ADC(2)-x transitions are positioned under the broad first experimental peak. Similarly, the broad second peak covers several transitions. After taking into account the translation, the position of the maximum of the second peak in the CVS-ADC(2)-x spectrum differs from the experimental one by 0.5 eV. Although the first peak in the CVS-ADC(2)-x spectrum also has a shoulder that is not seen in the experimental spectrum of Reynaud et al.,^87^ the difference in the spectral form can be tentatively explained by a slight overestimation of the second transition in the CVS-ADC(2)-x calculation: if the real transition is at a lower energy, the shoulder disappears. The shoulder also disappears from the simulated spectrum if a larger Gaussian broadening is used (shown in the Supporting Information). The differences between the positions of the CVS-ADC(2)-x and the measured peaks are 1.3 and 0.9 eV, respectively, for the experiment of Reynaud et al.,^87^ and −0.9 and −1.5 eV for the experiment of Bodeur and Esteva;^88^ see Table 3 for a full set of values. A tentative interpretation of the differences is that the CVS-ADC(2)-x method exhibits an error in the order of 1 eV around the sulfur K edge that overestimates all excitation energies, while the relative energies of the excitations are predicted more accurately, within an error of 0.5 eV. The CVS-ADC(2)-x data for all four molecules are given in the Supporting Information.

**Table 3:** Comparison of the Computed and Experimental Results in eV for the H_2_S Molecule^a^

	experiment	CVS-ADC(2)-x	CVS-CC2	CVS-CCSD	CVS-CC3
main edge (first peak)		2474.0	2475.2	2476.7	2474.4
Reynaud et al.^87^	2472.7	1.3	2.5	4.0	1.7
Bodeur and Esteva^88^	2473.1	0.9	2.1	3.6	1.3
second peak		2474.8	2475.8	2477.5	2476.7
Reynaud et al.^87^	2475.7	–0.9	0.1	1.8	1.0
Bodeur and Esteva^88^	2476.3	–1.5	–0.5	1.2	0.4

a Differences from the experimental values are shown in each column.

The translated TP-DFT spectrum of H_2_S is shown in Figure 1b. We find that the excitation energy of the first transition is overestimated by 0.5 eV for the experiment of Reynaud et al.^87^ and by 0.1 eV for the experiment by Bodeur and Esteva.^88^ The good agreement for the position of the first peak arises from the use of the ΔSCF procedure, which is well-known to reproduce rather accurate transition energies. However, excitations with small intensities, present at energies above 2473.6 eV on Figure 1b, lead to a TP-DFT spectrum that does not agree with experiment for H_2_S.

Table 3 and Figure 1–e show results of CC2, CCSD, and CC3 methods, respectively, for the H_2_S molecule. CC2 predicts only two peaks, as the first and second transitions are close in energy and the higher transitions are likewise spaced too closely together. CCSD predicts a larger spacing between the first and second transitions, splitting the first peak in two, while the higher-energy transitions are still grouped too closely together. At variance to CC2 and CCSD, in CC3, the first peak is generated by just one transition. Moreover, the near degeneracy of the higher excitations is also lifted. As a result, the CC3 method spectrum correctly reproduces all three broad experimental peaks, although the position of the second peak is still off by 0.5 eV. The positions of the first peaks, that is, the sulfur K edges, from CVS-ADC(2)-x and CVS-CC3, differ by 0.4 eV, being in good agreement, while the positions of the second peaks differ by a larger amount, 1.9 eV. The CVS-CC2, CVS-CCSD, and CVS-CC3 data for all four molecules are given in the Supporting Information.

### 3.2 SO_2_

The X-ray absorption spectrum of SO_2_ has been measured by Bodeur and Esteva^88^ and later by Reynaud et al.^87^ with an improved resolution. Reynaud et al.^87^ found the energy scale to be shifted by 0.6 eV from the previous experiment of Bodeur and Esteva^88^ and the ratios of the peak intensities to change as well. In addition, instead of measuring a peak with a shoulder as in Bodeur and Esteva,^88^ Reynaud et al.^87^ found the second and third resonances to be split by 0.8 eV.

**Figure 2:**
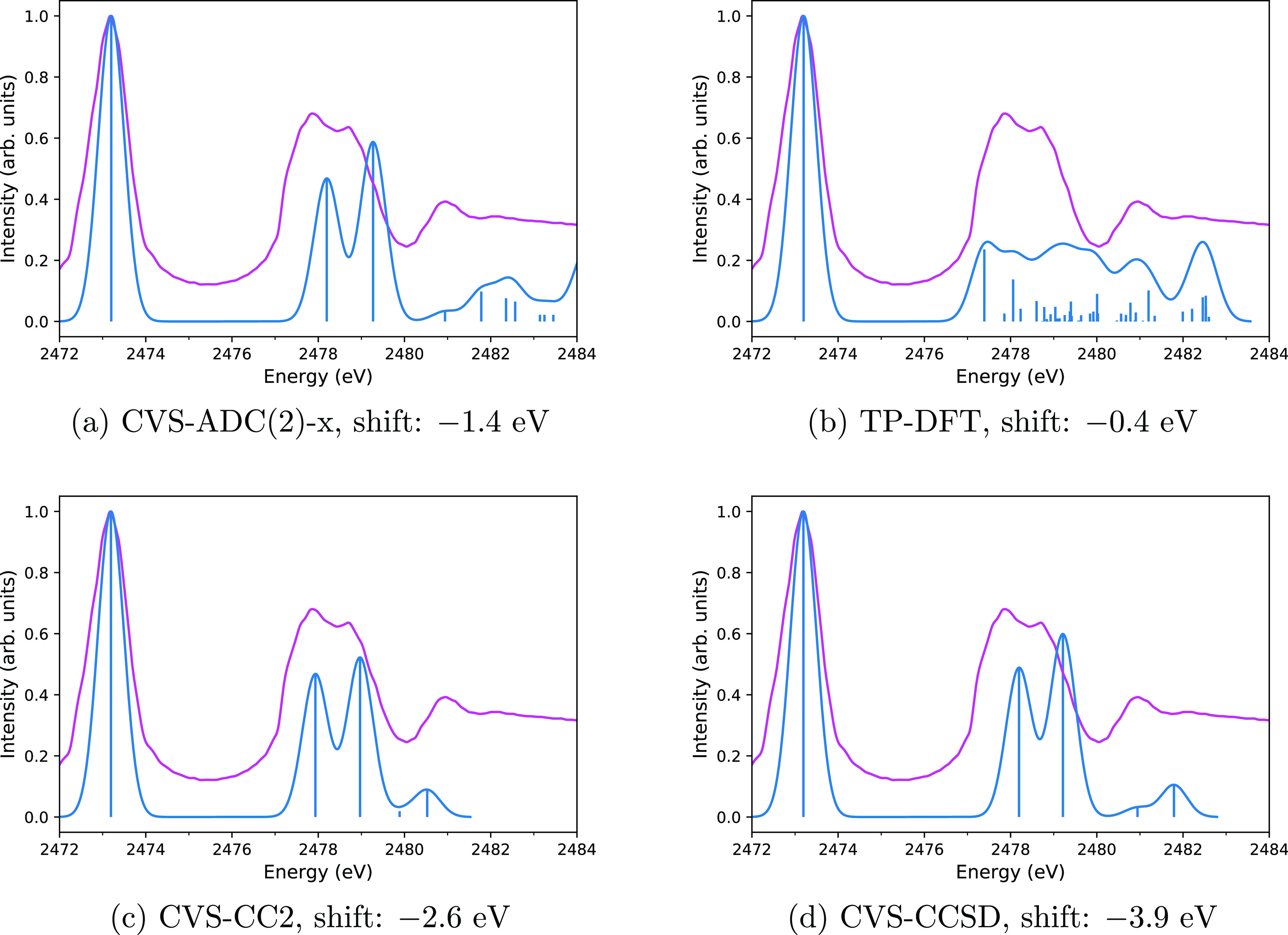
Comparison of computed (blue) and experimental (magenta) spectra of SO_2_ with the CVS-ADC(2)-x (a), TP-DFT (b), CVS-CC2 (c), and CVS-CCSD (d) methods. Computed spectra are translated by the amount shown in the captions to align the main peak to the experiment. Vertical lines represent the calculated spectra, whereas the curves are the theoretical spectra broadened for lifetime and detector resolution effects to mimic the shape of the experimental spectrum closely as possible. The experimental spectrum is adapted with permission from Reynaud et al.^87^ (copyright 1996 published by Elsevier B.V.).

The CVS-ADC(2)-x spectrum of SO_2_ is shown in Figure 2a. The values of the CVS-ADC(2)-x excitation energies are presented and compared to the experiments of Bodeur and Esteva^88^ and Reynaud et al.^87^ in Table 4. Despite the small disagreements between the two experiments, the findings from the data of Table 4 suggest that CVS-ADC(2)-x overestimates the transition energies. The relative peak positions are reproduced to good accuracy, however. Visual inspection of Figure 2a shows the CVS-ADC(2)-x and experimental spectrum to be in good agreement, while the TP-DFT spectrum (presented in Figure 2b) again shows considerable discrepancies from experiment due to the presence of many excitations for energies higher than 2477.4 eV. The positions of the second and third peaks in the CVS-ADC(2)-x spectrum of Figure 2a differ by 0.3 and 0.4 eV from the corresponding experimental peaks. The position of the fifth transition is off by 1 eV from the fourth experimental peak at 2481 eV. The fourth peak appears to cover several computed excitations.

**Table 4:** Comparison of the Computed and Experimental Results in eV for the SO_2_ Molecule^a^

	experiment	CVS-ADC(2)-x	CVS-CC2	CVS-CCSD
main edge (first peak)		2474.6	2475.8	2477.1
Reynaud et al.^87^	2473.2	1.4	2.6	3.9
Bodeur and Esteva^88^	2473.8	0.8	2.0	3.3
second peak		2479.6	2480.5	2482.1
Reynaud et al.^87^	2477.9	1.7	2.6	4.2
Bodeur and Esteva^88^	2478.4	1.2	2.1	3.7
third peak		2480.7	2481.6	2483.1
Reynaud et al.^87^	2478.7	2.0	2.9	4.4
Bodeur and Esteva^88^	2478.9	1.8	2.7	4.2

a Differences from the experimental values are shown in each column.

Although the TP-DFT spectrum (shown in Figure 2b) does not agree with the experiment as a whole, the position of the sulfur K edge from the ΔSCF calculation is again in excellent agreement with the experimental value: the position of the main peak is overestimated by 0.4 eV compared to the experiment by Reynaud et al.^87^ and underestimated by 0.2 eV compared to the experiment by Reynaud et al.^87^

The CVS-CC2 and CVS-CCSD spectra for SO_2_ are shown in Figure 2c,d, respectively. Although the relative position and form of the broad second peak are reproduced better by CVS-CC2 than CVS-CCSD, as seen in Figure 2c,d, both the CVS-CC2 and CVS-CCSD spectra are close to the measured spectrum. The results for CVS-CC2 and CVS-CCSD are summarized in Table 4.

### 3.3 OCS

The X-ray spectrum of OCS has been measured by Perera and LaVilla^89^ as well as by Nenner et al.^90^ Even though the experiment by Perera and LaVilla^89^ is older than the one by Nenner et al.,^90^ its spectrum is at a higher resolution than the one of the newer experiment of ref (90). Therefore, we decided to analyze the calculations with respect to the experiment by Perera and LaVilla.^89^

**Figure 3:**
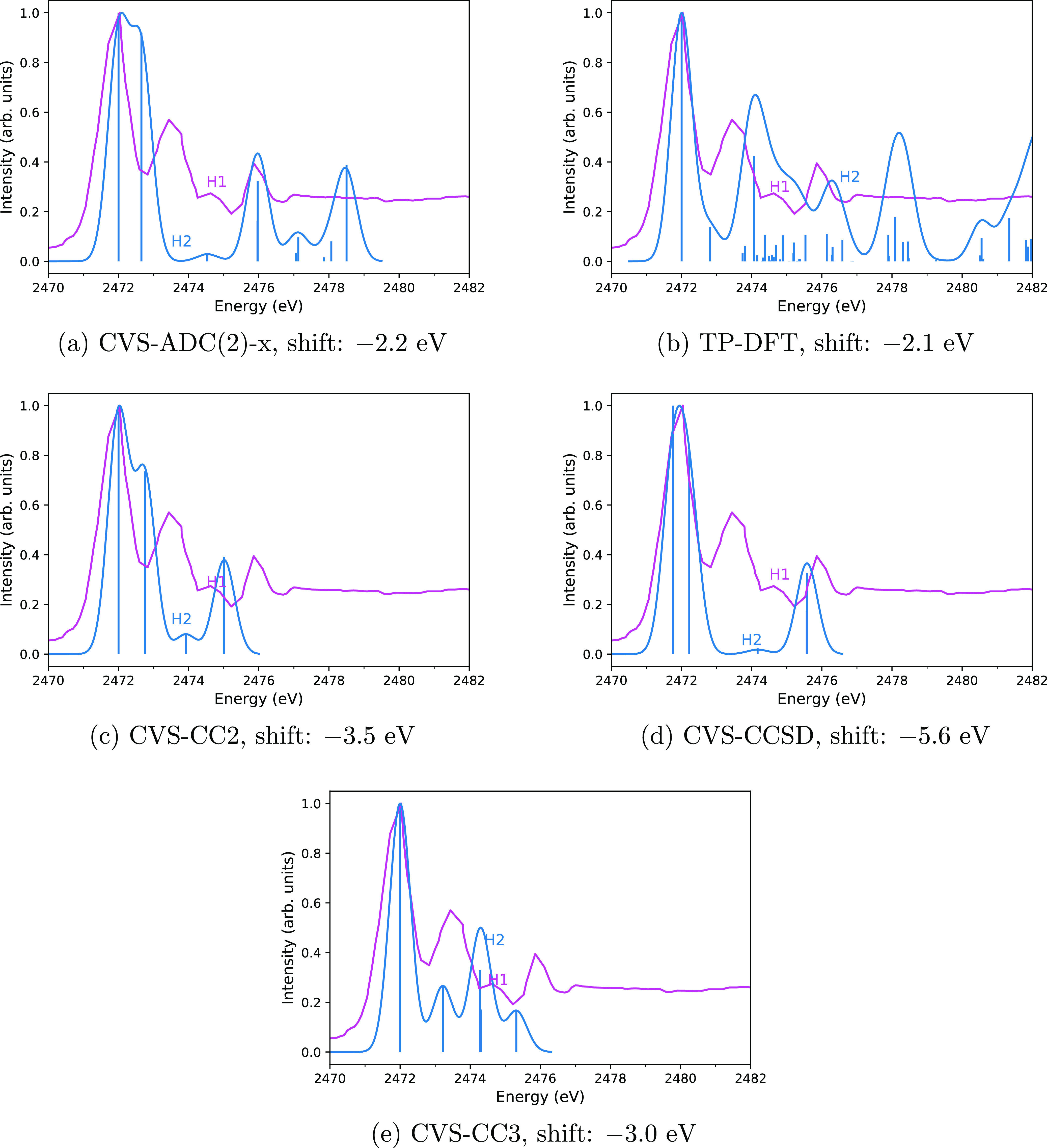
Comparison of computed (blue) and experimental (magenta) spectra of OCS with the CVS-ADC(2)-x (a), TP-DFT (b), CVS-CC2 (c), CVS-CCSD (d), and CVS-CC3 (e) methods. Computed spectra are translated by the amount shown in the captions to align the main peak to the experiment. Vertical lines represent the calculated spectra, whereas the curves are the theoretical spectra broadened for lifetime and detector resolution effects to mimic the shape of the experimental spectrum closely as possible. The experimental spectrum of OCS adapted with permission from Perera and LaVilla^89^ (copyright 1984 published by AIP). The letter H was used in the measured spectra by Perera and LaVilla^89^ to label the third peak with a small intensity.

The CVS-ADC(2)-x spectrum for OCS is shown in Figure 3a. A visual inspection shows that even though the first computed transition differ from the experimental value by 2.2 eV (as presented in Table 5), the form of the spectrum, as interpreted from the stick transitions, agrees well with the experiment, since all three major peaks of the experiment are visible in the calculation. The CVS-ADC(2)-x method even reproduces the small measured peak labeled “H” in ref (89). After the alignment, the fourth computed and experimental peaks turn out to be at the same position. The small peaks (labeled “H1” and “H2”) are also close to each other. However, the second transition in CVS-ADC(2)-x is 0.7 eV too close to the first one, compared to experiment. The intensity of the second transition is also much too high compared to experiment.

**Table 5:** Comparison of the Computed and Experimental Results in eV for the OCS Molecule^a^

	experiment	CVS-ADC(2)-x	CVS-CC2	CVS-CCSD	CVS-CC3
main edge (first peak)		2474.2	2475.5	2477.4	2475.0
Nenner et al.^90^	2471.2	3.0	4.3	6.2	3.8
Perera and LaVilla^89^	2472.0	2.2	3.5	5.4	3.0
second peak		2474.9	2476.2	2477.8	2476.2
Nenner et al.^90^	2472.7	2.2	3.5	5.1	3.5
Perera and LaVilla^89^	2473.4	1.5	2.8	4.4	2.8
third peak		2478.2	2478.5	2481.2	2478.3
Nenner et al.^90^	2475.2	3.0	3.3	6.0	3.1
Perera and LaVilla^89^	2475.9	2.3	2.6	5.3	2.4
peak “H”		2476.8	2477.4	2479.8	2477.3
Perera and LaVilla^89^	2474.6	2.2	2.8	5.2	2.7

a Differences from the experimental values are shown in each column.

The TP-DFT spectrum shown in Figure 3b once again does not agree with the experimental data due to the presence of many excitations of small intensities. However, four broad computed peaks are visible in Figure 3b, including the peak H.

The agreement of the peak positions of the CVS-ADC(2)-x calculations and the two experiments is analyzed in detail in Table 5. Despite an error of a few eV—larger than those observed above for H_2_S and SO_2_ in Tables 3 and 4, respectively—the relative positions of the majority of CVS-ADC(2)-x peaks are rather accurate. The exception is the second computed peak. Like the CVS-ADC(2)-x calculation, the ΔSCF calculation on OCS also shows a larger discrepancy for the sulfur K edge: the position of the main peak is overestimated by 2.9 eV for the experiment by Nenner et al.^90^ and by 2.1 eV for the experiment by Perera and LaVilla.^89^

The CVS-CC2, CVS-CCSD, and CVS-CC3 spectra are shown in Figure 3c–e, respectively. All four experimental peaks are reproduced by all three CC methods: the third experimental peak (labeled “H” in the experiment^89^ and “H1” in the experimental spectrum in Figure 3) is also visible on the computed spectra (labeled “H2”). Like CVS-ADC(2)-x, CVS-CC2, and CVS-CCSD underestimate the energy of the second excitation and overestimate its intensity compared to experiment, CVS-CC3, in turn, also underestimates the energy of the second transition and furthermore underestimates its intensity considerably. The intensity of the third peak is underestimated by CVS-CC2 and CVS-CCSD and overestimated by CVS-CC3. Its position relative to the sulfur K edge is underestimated by all three CC methods. The intensity of the fourth peak is reproduced by CVS-CC2 and CVS-CCSD, while it is underestimated by CVS-CC3. Its position is reproduced by CVS-CCSD, while it is underestimated by CVS-CC2 and CVS-CC3. A summary of the CVS-CC2, CVS-CCSD, and CVS-CC3 results is given in Table 5.

### 3.4 CS

**Figure 4:**
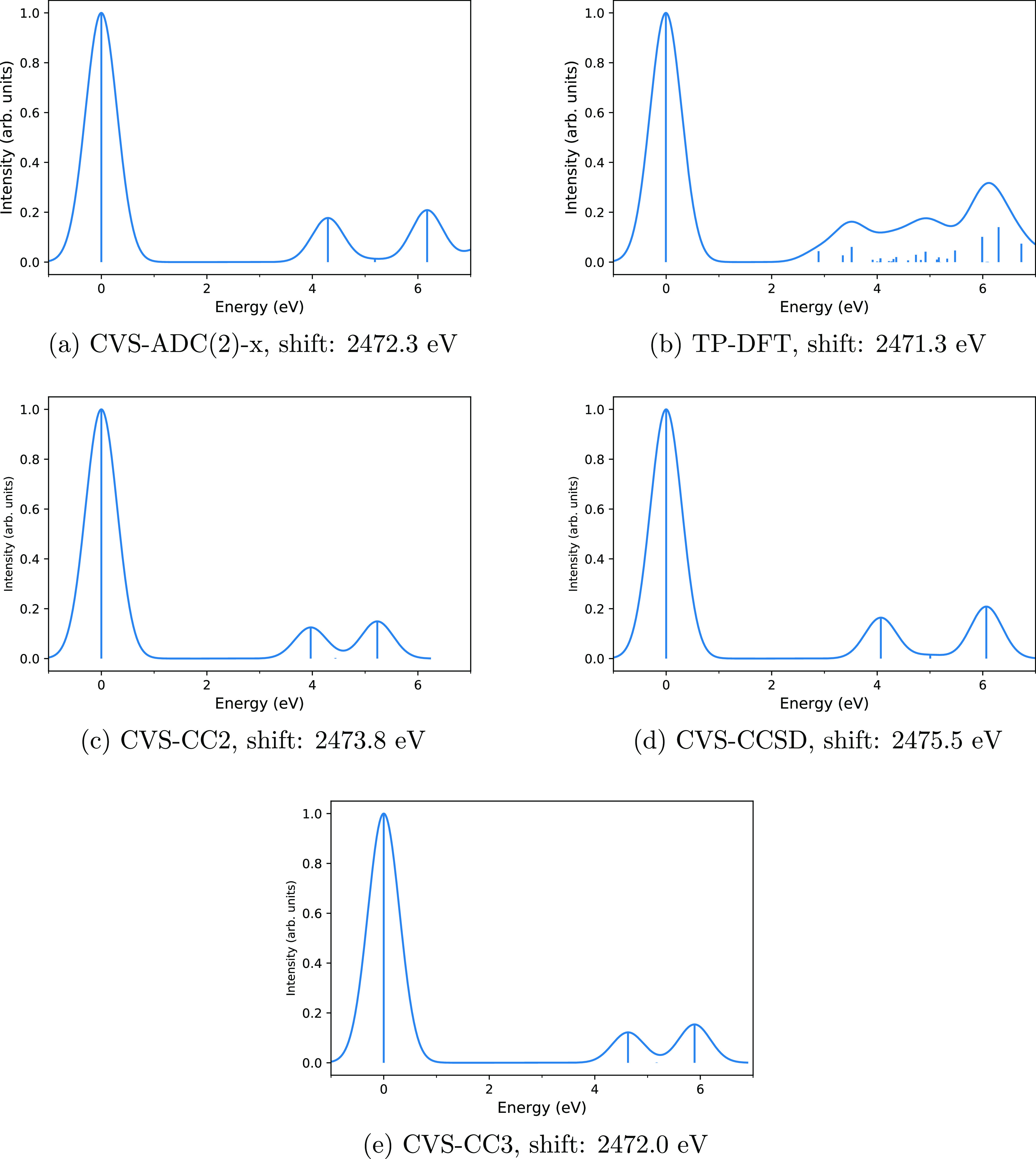
Computed spectra of CS with the CVS-ADC(2)-x (a), TP-DFT (b), CVS-CC2 (c), CVS-CCSD (d), and CVS-CC3 (e) methods. Computed spectra are shown translated to the corresponding sulfur K edge. Vertical lines represent the calculated spectra, whereas the curves are the theoretical spectra broadened for lifetime and detector resolution effects to mimic the possible shape of the experimental spectrum that is not available.

As stated above, we are not aware of the measured spectrum for CS. The spectra computed with the CVS-ADC(2)-x, TP-DFT, CVS-CC2, CVS-CCSD, and CVS-CC3 methods are shown in Figure 4b,a,c–e, respectively. The CVS-ADC and CVS-CC spectra again agree qualitatively; the results are summarized in Table 6. Especially, the CVS-CC3 and CVS-ADC(2)-x spectra in Figure 4e,a are in excellent agreement. The positions of the first and second peaks in the CVS-ADC(2)-x and CVS-CC3 calculations differ by 0.3 and 0.4 eV, respectively. The position of the third peak is the same in both methods. These differences can be contrasted to a recent study by Myhre et al.^91^ that compared CVS-CCSD, CVS-CC3, and CVS-ADC(2)-x spectra for the nitrogen K edge in N_2_ to synchrotron experiments. Myhre et al. found the position of the first peak to be 0.13 eV which is too high in CVS-CC3, 0.91 eV which is too high in CVS-CCSD, and 1.65 eV which is too low in CVS-ADC(2)-x, that is, a difference of 1.78 eV between the CC3 and ADC(2)-x predictions. The excellent level of agreement between our CVS-CC3 and CVS-ADC(2)-x calculations for the sulfur K edge in CS suggests that the computed spectra are reliable.

**Table 6:** Position of First Peak and Relative Positions of Second and Third Peaks in eV from CVS-ADC(2)-x and CVS-CC Calculations for the CS Molecule

	CVS-ADC(2)-x	CVS-CC2	CVS-CCSD	CVS-CC3
main edge (first peak)	2472.3	2473.8	2475.6	2472.0
second peak, relative to edge	4.3	3.9	4.0	4.7
third peak, relative to edge	5.2	4.4	5.0	5.2

TP-DFT once again disagrees with the CVS-ADC(2)-x and CVS-CC calculations, producing several transitions above 2474 eV that do not appear in the CVS-ADC(2)-x or CVS-CC spectra. Because the CVS-ADC and CVS-CC spectra agreed well with the experiment for H_2_S, SO_2_, and OCS, this disagreement suggests that the TP-DFT model is not suitable for accurate modeling of the sulfur K edge spectra of CS.

## 4 Discussion

We calculated sulfur K edge X-ray spectra of H_2_S, SO_2_, OCS, and CS using the ADC method CVS-ADC(2)-x; the CVS-CC2, CVS-CCSD, and CVS-CC3 CC methods; and the TP approximation of DFT with the ωB97M-V range-separated functional. Augmented, uncontracted quadruple-ζ basis sets were used in this work, as we found them to afford sufficiently converged spectra at all the aforementioned levels of theory.

It was found that the ΔSCF method produces excellent agreement with experiment for H_2_S and SO_2_: the differences were 0.5 eV or less. However, comparison to the experiment showed that the TP-DFT method does not yield a reliable description for the XANES spectra of the studied four molecules.

In contrast, the CVS-ADC(2)-x and CVS CC spectra were found to be in qualitatively good agreement with experiments for H_2_S, SO_2_, and OCS. The shapes of the CVS-ADC(2)-x and CC spectra were found to be overall correct, and even though the excitation energies were found to be slightly overestimated, the relative energies of the excitations were found to be reproduced more accurately. An excellent agreement between the CVS-CC3 and CVS-ADC(2)-x spectra was found for CS, for which we are not aware of a measured spectrum, suggesting that our predictions are accurate. The predictions of CVS-ADC(2)-x, CVS-CC2, and CVS-CCSD for the XANES spectrum of SO_2_ are also in outstanding agreement.

In contrast, there are noticeable differences between the spectra reproduced by CVS-CC3 and CVS-ADC(2)-x in the cases of H_2_S and OCS. For both of these molecules, neither method is sufficient for a quantitative reproduction of the experimental XANES spectrum. We expect that proceeding to a higher level of CC theory, such as the CCSDT or CCSDTQ method, would allow in-detail reproduction of the intensities and relative positions of the peaks seen in the experiment. Similarly, CVS-ADC results will generally improve if higher levels of ADC are employed.

The agreement found in this work between the positions of the peaks from CVS-ADC(2)-x calculations and experiment for H_2_S, SO_2_, and OCS at the sulfur K edge is less remarkable than that reported for CVS-ADC studies at the carbon, oxygen, and nitrogen K edges in several molecules.^44^ The case of the OCS molecule is plausibly explained by errors in the experiment, while the disagreements in the order of 1 eV for the sulfur K edge in H_2_S and SO_2_ are much smaller than the disagreements in CVS-ADC calculations for sulfur reported so far to the best of our knowledge. In the only work we are aware of, in which CVS-ADC(2)-x calculations of the sulfur K edge have been presented, Wenzel et al.^44^ found the ADC(2)-x/6-311++G** sulfur K edge in bithiophene, (C_4_H_3_S)_2_, to be underestimated by 5.1 eV compared to experiment. However, Wenzel et al.^44^ did not include a relativistic correction: with a typical relativistic correction for sulfur of (7–8) eV, their sulfur K edge would be overestimated by (2–3) eV. To confirm the validity of the use of a semiempirical relativistic correction, we calculated a sulfur K edge of 2466.3 eV in H_2_S with the nonrelativistic CVS-ADC(2)-x/un-aug-pc-3 method, which is 7.7 eV lower than the corresponding relativistic value of 2474.0 eV. This difference is explained almost entirely by the typical relativistic correction of (7–8) eV: indeed, as discussed in Section 2.3, the sulfur 1s shift in H_2_S at the HF/un-aug-pc-3 level of theory (upon which the ADC method builds) is 7.9 eV; thus, the error of the semiempirical approach compared to a full relativistic calculation within the X2C method is just +0.2 eV for the H_2_S molecule.

Because the use of a relativistic shift thus appears to be justified, our CVS-ADC(2)-x values for the sulfur K edge in H_2_S and SO_2_ are in better agreement with the experiment than the one for bithiophene computed in ref (44). The calculation of Wenzel et al.^44^ was limited by a small contracted basis set that is known to be ill-behaving;^92,93^ the reason for our improved results is likely the application of a large uncontracted basis set, which enables the use of the CVS-ADC(2)-x method for accurate modeling of sulfur K edge spectra.

The experimental results for H_2_S, SO_2_, and OCS are rather old, and comparisons of the experiments with the spectra calculated in this work shows that new measurements with the now possible higher resolutions are necessary to resolve the discrepancies between calculations and experiments. The differences between the absolute energy scales of the experiments are large, suggesting there is room for new experiments. The difference between the calculated and experimental excitation energies was found to be especially large for OCS in all the CVS-ADC(2)-x, CVS-CC, and the ΔSCF calculations, suggesting that experiments should be revisited especially for this molecule.

## 5 Conclusions and Perspectives

The initial motivation for this study was to generate sulfur molecule spectra that would allow future X-ray telescopes to identify and measure the amounts of sulfur molecules in astrophysical environments. However, we find that it is too early for this goal: the agreement between theoretical and experimental spectra, as well as between different experiments, is not at the level required by the next generation of X-ray telescopes. XRISM and Athena, set to launch in 2022 and 2031, respectively, will have spectral resolutions of 5 and 2.5 eV, respectively. Lynx, if selected for further development, would launch in 2036 and have 0.5 eV resolution. More laboratory and computational studies are thereby solely needed for the further development of X-ray astrochemistry.^94^

We hope that future high-resolution X-ray facilities and developments in computing and measuring X-ray spectra will establish X-ray methods as standard tools in detecting and identifying molecules and dust materials in space. Such work requires large, easily accessible databases of reference spectra. A database of core excitations for gas phase molecules^95^ already exists but is outdated and is oriented more toward organic chemistry and related applications than toward the demands of astrophysical applications. As updated, high resolution spectral databases similar to the PAH IR spectral database^96^ should be prepared for the X-ray regime in anticipation of significantly increased future demand.

## Supporting Information Available

Molecular structures for H_2_S, SO_2_, OCS, and CS; CVS-ADC(2)-x/un-aug-cc-pV(Q+d)Z spectra; CVS-ADC(2)-x/un-aug-pc-3 spectra with a smaller Gaussian broadening; CVS-ADC(2)-x/un-aug-pc-3 spectrum for H_2_S with a larger Gaussian broadening; CVS-ADC(2)-x/un-aug-pc-3 spectra with Lorentzian broadening; CVS-ADC(2)-x/un-aug-pc-3 stick spectra for H_2_S and CS, also includes data for the un-daug-pc-3 and un-taug-pc-3 basis sets; CVS-ADC(2)-x/un-aug-pc-3 stick spectra for SO_2_ and OCS; basis set convergence of CC calculations for H_2_S, SO_2_, OCS, and CS; CVS-CC2/un-aug-pc-3 stick spectra for H_2_S, SO_2_, OCS, and CS, also includes data for the un-daug-pc-3 basis set; and CVS-CCSD/un-aug-pc-3 stick spectra for H_2_S, SO_2_, OCS, and CS; CVS-CC3/un-aug-pc-3 stick spectra for H_2_S and CS (PDF)
