# Supplementary material for: Sulfur Molecules in Space by X-rays: A Computational Study
Source: ACS Earth Space Chem. 2021 Feb 24;5(3):436–48. doi: 10.1021/acsearthspacechem.0c00238 (PMC8028330; doi:10.1021/acsearthspacechem.0c00238)
Supplement: Supplementary file 1 — sp0c00238_si_001.pdf [file sp0c00238_si_001.pdf]

Supporting Information: Sulfur molecules in space by X-rays: a computational study

Goranka Bilalbegović,[*,†] Aleksandar Maksimović,[‡] Lynne A. Valencic,[¶,§] and Susi Lehtola[*,‖,⊥]

†*Department of Physics, Faculty of Science, University of Zagreb, Bijenička cesta 32, 10000 Zagreb, Croatia*

‡*Center of Excellence for Advanced Materials and Sensing Devices, Rudjer Bošković Institute, Bijenička cesta 54, 10000 Zagreb, Croatia*

¶*NASA Goddard Space Flight Center, Greenbelt, MD 20771, USA*

§*The Johns Hopkins University, Department of Physics & Astronomy, 366 Bloomberg Center, 3400 N. Charles St., Baltimore, MD 21218, USA*

‖*Department of Chemistry, P.O. Box 55 (A. I. Virtasen aukio 1), FI-00014 University of Helsinki, Finland*

⊥*Present address: Molecular Sciences Software Institute, Blacksburg, Virginia 24061, USA*

E-mail: goranka.bilalbegovic@gmail.com; susi.lehtola@alumni.helsinki.fi

Contents:

- Molecular structures for H_2S, SO_2, OCS, and CS: tables S1, S2, S3 and S4, respectively
- CVS-ADC(2)-x/un-aug-cc-pV(Q+d)Z spectra: figure S1
- CVS-ADC(2)-x/un-aug-pc-3 spectra with a smaller Gaussian broadening: figure S2
- CVS-ADC(2)-x/un-aug-pc-3 spectrum for H_2S with a larger Gaussian broadening: figure S3
- CVS-ADC(2)-x/un-aug-pc-3 spectra with Lorentzian broadening: figure S4
- CVS-ADC(2)-x/un-aug-pc-3 stick spectra for H_2S and CS: tables S5 and S6, respectively. Also includes data for the un-daug-pc-3 and un-taug-pc-3 basis sets
- CVS-ADC(2)-x/un-aug-pc-3 stick spectra for SO_2 and OCS: tables S7 and S8, respectively
- Basis set convergence of CVS coupled cluster calculations for H_2S, SO_2, OCS, and CS: tables S9, S10, S11 and S12, respectively
- CVS-CC2/un-aug-pc-3 stick spectra for H_2S, SO_2, OCS, and CS: tables S13, S14, S15 and S16, respectively. Also includes data for the un-daug-pc-3 basis set
- CVS-CCSD/un-aug-pc-3 stick spectra for H_2S, SO_2, OCS, and CS: tables S17, S18, S19 and S20, respectively.
- CVS-CC3/un-aug-pc-3 stick spectra for H_2S, CS and OCS: tables S21, S22 and S23, respectively.

Table S1: Molecular structure for H_2S.

atom	x (Å)	y (Å)	z (Å)
S	-0.00001	-0.00001	0.10328
H	-0.00000	0.96537	-0.82371
H	0.00001	-0.96537	-0.82371

Table S2: Molecular structure for SO_2.

atom	x (Å)	y (Å)	z (Å)
S	0.00000	0.00002	0.36551
O	0.00000	1.23148	-0.36386
O	-0.00000	-1.23149	-0.36387

Table S3: Molecular structure for OCS.

atom	x (Å)	y (Å)	z (Å)
S	0.00000	0.00000	1.03492
C	0.00000	0.00000	-0.52726
O	0.00000	0.00000	-1.67800

Table S4: Molecular structure for CS.

atom	x (Å)	y (Å)	z (Å)
S	0.00000	0.00000	-0.41528
C	0.00000	0.00000	1.10846

Table S5: CVS-ADC(2)-x stick spectrum for H_2S, computed in the un-aug-pc-3 basis set and its doubly (daug) and triply (taug) augmented counterparts.

un-aug-pc-3		un-daug-pc-3		un-taug-pc-3	
E (eV)	I (arb. units)	E (eV)	I (arb. units)	E (eV)	I (arb. units)
2474.006	0.0062	2474.006	0.0062	2474.006	0.0062
2474.764	0.0031	2474.764	0.0031	2474.764	0.0031
2477.529	0.0011	2477.501	0.0010	2477.501	0.0010
2477.586	0.0005	2477.580	0.0005	2477.580	0.0005
2477.619	0.0007	2477.585	0.0006	2477.585	0.0006
2477.746	0.0017	2477.710	0.0016	2477.710	0.0016
2478.28	0.0000	2478.196	0.0000	2478.196	0.0000
2478.595	0.0000	2478.372	0.0000	2478.372	0.0000
2478.738	0.0000	2478.495	0.0000	2478.494	0.0000
2479.032	0.0000	2478.645	0.0000	2478.643	0.0000

Table S6: CVS-ADC(2)-x stick spectrum for CS, computed in the un-aug-pc-3 basis set and its doubly (daug) and triply (taug) augmented counterparts.

un-aug-pc-3		un-daug-pc-3		un-taug-pc-3	
E (eV)	I (arb. units)	E (eV)	I (arb. units)	E (eV)	I (arb. units)
2472.284	0.0048	2472.283	0.0048	2472.283	0.0048
2472.284	0.0048	2472.283	0.0048	2472.283	0.0048
2476.578	0.0017	2476.577	0.0017	2476.576	0.0017
2477.470	0.0001	2477.450	0.0001	2477.450	0.0001
2478.459	0.0010	2478.447	0.0009	2478.447	0.0009
2478.459	0.0010	2478.447	0.0009	2478.447	0.0009
2479.315	0.0002	2479.142	0.0000	2479.139	0.0000
2479.454	0.0000	2479.142	0.0000	2479.139	0.0000
2479.454	0.0000	2479.154	0.0003	2479.153	0.0003
2479.695	0.0006	2479.483	0.0002	2479.476	0.0001

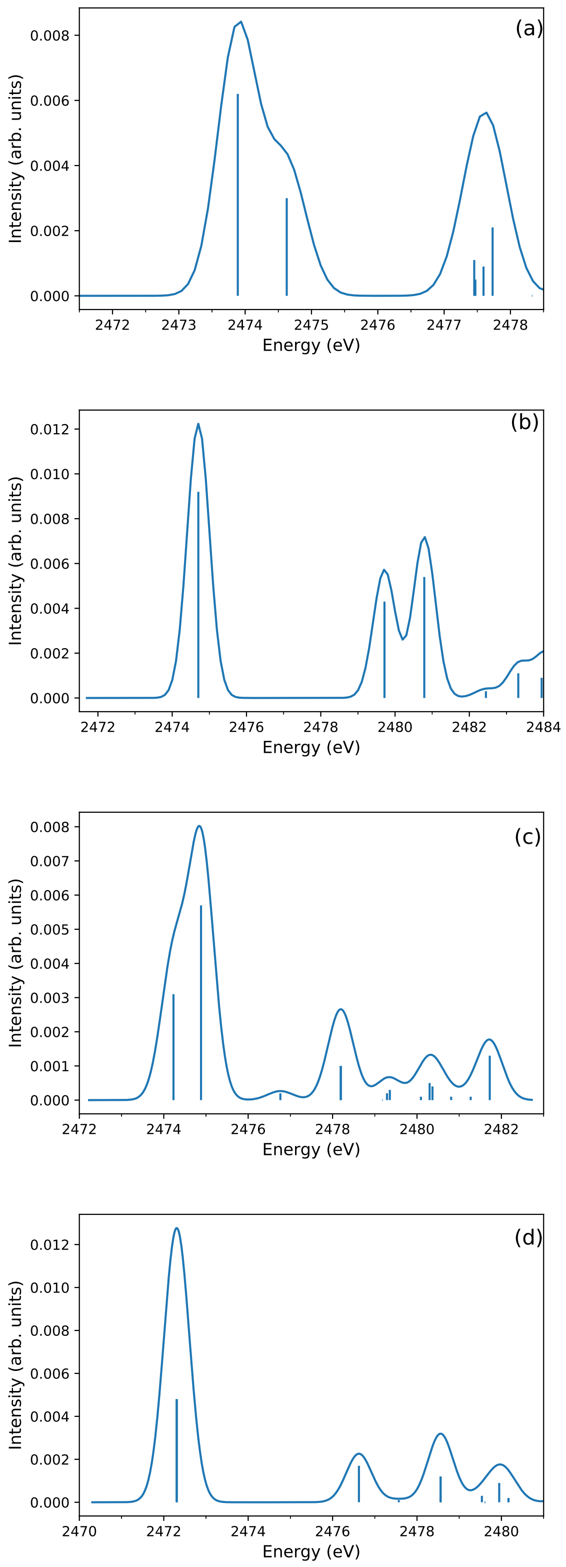

Figure S1: CVS-ADC(2)-x/un-aug-cc-pV(Q+d)Z spectra for (a) H_2S, (b) SO_2, (c) OCS, (d) CS. Vertical lines represent the calculated spectra, whereas the curves are the theoretical spectra broadened with Gaussian functions to simulate lifetime and detector resolution effects. The broadening parameter is 0.3 eV.

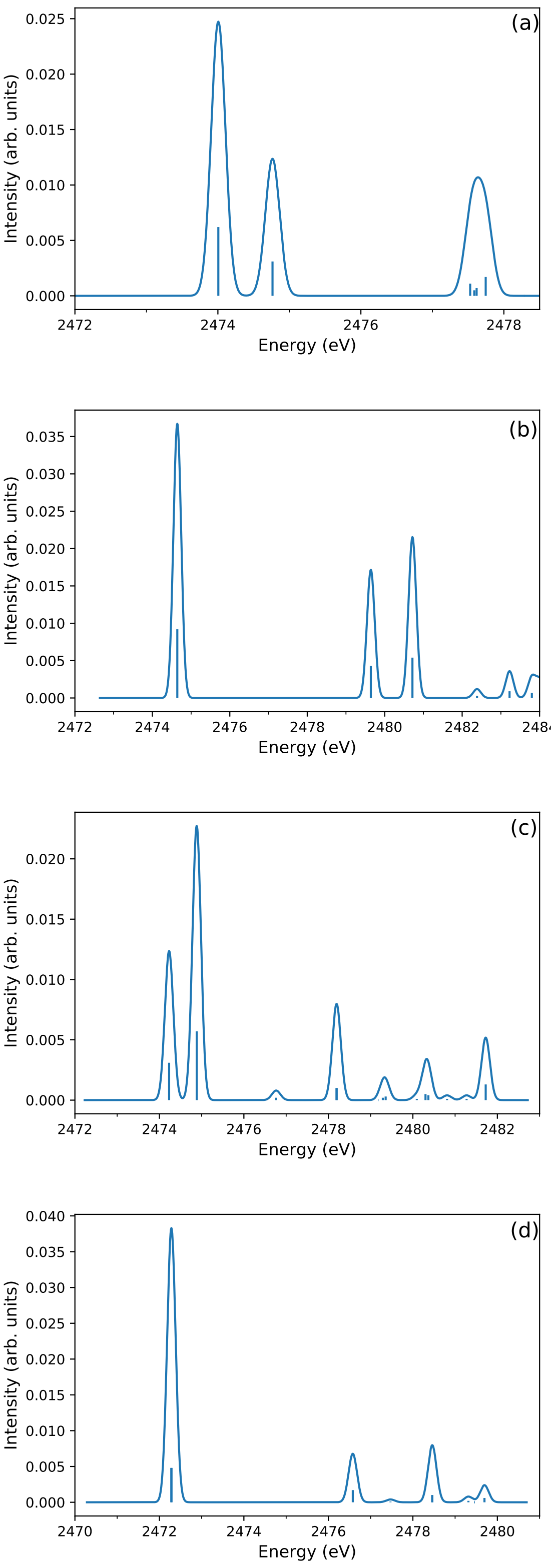

Figure S2: The CVS-ADC(2)-x/un-aug-pc-3 spectra with a small Gaussian broadening of 0.1 eV for (a) H_2S, (b) SO_2, (c) OCS, (d) CS. Vertical lines represent the calculated spectra, whereas the curves are the theoretical spectra broadened to simulate lifetime and detector resolution effects.

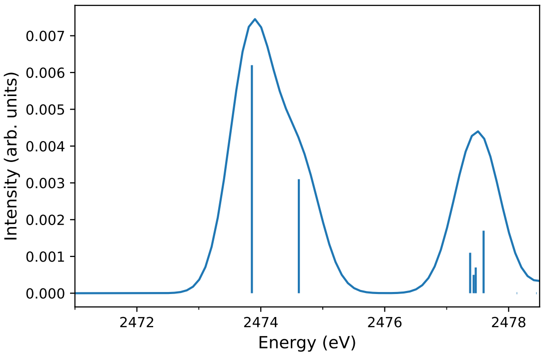

Figure S3: CVS-ADC(2)-x/un-aug-pc-3 spectrum of H_2S using the un-aug-pc-3 basis and a Gaussian broadening of 0.35 eV, showing that the shoulder of the first peak disappears. Vertical lines represent the calculated spectra, whereas the curves are the theoretical spectrum broadened to simulate lifetime and detector resolution effects.

Table S7: CVS-ADC(2)-x/un-aug-pc-3 stick spectrum for SO_2.

E (eV)	I (arb. units)
2474.644	0.0092
2479.642	0.0043
2480.716	0.0054
2482.384	0.0003
2483.224	0.0009
2483.800	0.0007
2484.011	0.0006
2484.586	0.0002
2484.691	0.0002
2484.897	0.0002
2484.902	0.0000
2485.030	0.0000
2485.475	0.0006
2485.762	0.0019
2486.085	0.0001
2486.527	0.0004
2486.581	0.0001
2486.643	0.0009
2486.907	0.0000
2487.085	0.0001

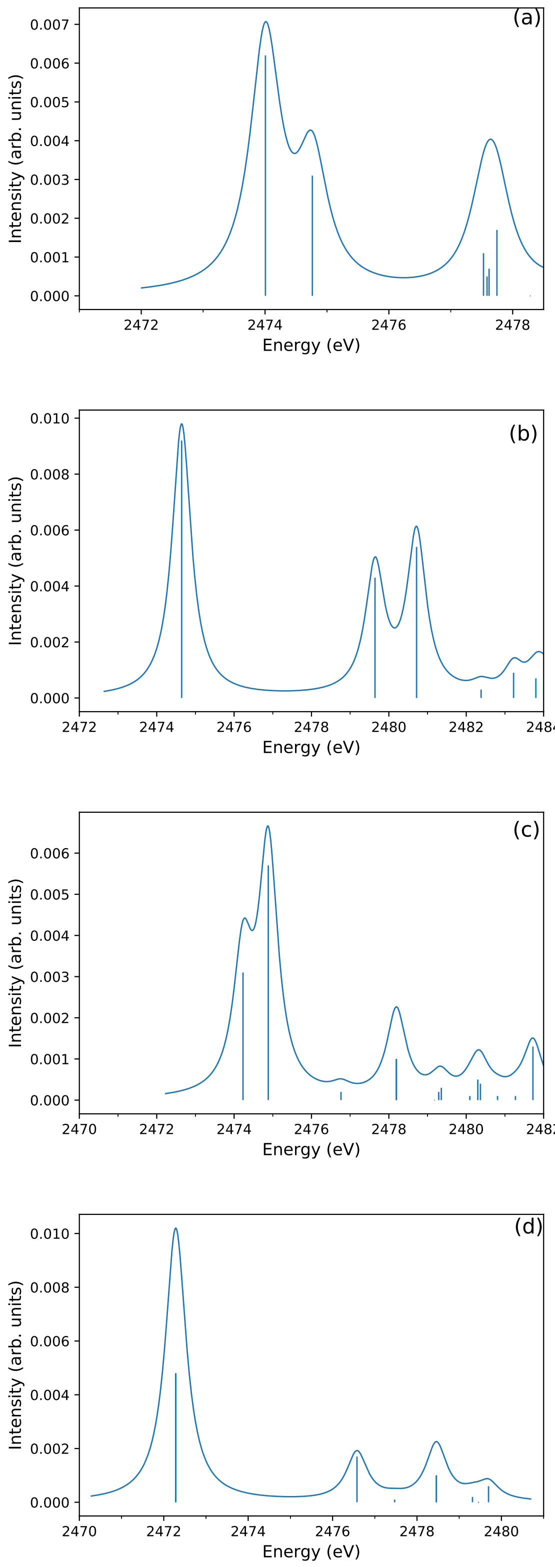

Figure S4: CVS-ADC(2)-x/un-aug-pc-3 spectrum for (a) H_2S, (b) SO_2, (c) OCS, (d) CS. Vertical lines represent the calculated spectra, whereas the curves are the theoretical stick spectra (ω_i, I_i) broadened with the Lorentzian function $I(\omega) = \sum_i \gamma I_i / [\pi[(\omega - \omega_i)^2 + \gamma^2]]$ to simulate lifetime and detector resolution effects. The broadening is $\gamma = 0.3$ eV.

Table S8: CVS-ADC(2)-x/un-aug-pc-3 stick spectrum for OCS.

E (eV)	I (arb. units)
2474.230	0.0031
2474.230	0.0031
2474.883	0.0057
2476.763	0.0002
2478.193	0.0010
2478.196	0.0010
2478.196	0.0010
2479.175	0.0000
2479.175	0.0000
2479.289	0.0002
2479.356	0.0003
2479.356	0.0003
2480.093	0.0001
2480.299	0.0005
2480.367	0.0004
2480.367	0.0004
2480.809	0.0001
2481.272	0.0001
2481.272	0.0001
2481.724	0.0013

Table S9: Basis set convergence of coupled cluster results for the sulfur K edge of H_2S.

basis	CVS-CC2 (eV)	CVS-CCSD (eV)	CVS-CC3 (eV)
un-aug-pc-0	2477.0	2478.6	2475.8
un-aug-pc-1	2476.0	2477.4	2474.8
un-aug-pc-2	2475.5	2476.9	2474.4
un-aug-pc-3	2475.2	2476.7	2474.4

Table S10: Basis set convergence of coupled cluster results for the sulfur K edge of SO_2.

basis	CVS-CC2 (eV)	CVS-CCSD (eV)
un-aug-pc-0	2478.4	2479.4
un-aug-pc-1	2477.0	2478.2
un-aug-pc-2	2476.1	2477.4
un-aug-pc-3	2475.8	2477.1

Table S11: Basis set convergence of coupled cluster results for the sulfur K edge of OCS.

basis	CVS-CC2 (eV)	CVS-CCSD (eV)
un-aug-pc-0	2477.5	2479.4
un-aug-pc-1	2476.2	2478.1
un-aug-pc-2	2475.7	2477.6
un-aug-pc-3	2475.5	2477.4

Table S12: Basis set convergence of coupled cluster results for the sulfur K edge of CS.

basis	CVS-CC2 (eV)	CVS-CCSD (eV)	CVS-CC3 (eV)
un-aug-pc-0	2475.7	2477.5	2474.0
un-aug-pc-1	2474.4	2476.2	2472.7
un-aug-pc-2	2474.0	2475.8	2472.3
un-aug-pc-3	2473.8	2475.6	2472.0

Table S13: CVS-CC2 stick spectrum for H_2S, computed in the un-aug-pc-3 basis set and its doubly (daug) augmented counterpart. Relativistic shift is not included.

un-aug-pc-3		un-daug-pc-3	
E (eV)	I (arb. units)	E (eV)	I (arb. units)
2467.315	0.0055	2467.315	0.0055
2467.866	0.0023	2467.866	0.0023
2470.039	0.0003	2470.019	0.0003
2470.134	0.0012	2470.068	0.0010
2470.220	0.0004	2470.154	0.0003
2470.260	0.0012	2470.180	0.0010

Table S14: CVS-CC2 stick spectrum for SO_2, computed in the un-aug-pc-3 basis set and its doubly (daug) augmented counterpart. Relativistic shift is not included.

un-aug-pc-3		un-daug-pc-3	
E (eV)	I (arb. units)	E (eV)	I (arb. units)
2467.893	0.0082	2467.892	0.0082
2472.634	0.0038	2472.632	0.0038
2473.670	0.0043	2473.669	0.0043
2474.587	0.0002	2474.535	0.0001
2475.227	0.0007	2475.206	0.0007

Table S15: CVS-CC2 stick spectrum for OCS, computed in the un-aug-pc-3 basis set and its doubly (daug) augmented counterpart. Relativistic shift is not included.

un-aug-pc-3		un-daug-pc-3	
E (eV)	I (arb. units)	E (eV)	I (arb. units)
2467.577	0.0029	2467.576	0.0029
2467.577	0.0029	2467.576	0.0029
2468.330	0.0043	2468.329	0.0043
2469.497	0.0005	2469.480	0.0005
2470.589	0.0009	2470.557	0.0008
2470.590	0.0007	2470.558	0.0006
2470.590	0.0007	2470.558	0.0006

Table S16: CVS-CC2 stick spectrum for CS, computed in the un-aug-pc-3 basis set and its doubly (daug) augmented counterpart. Relativistic shift is not included.

un-aug-pc-3		un-daug-pc-3	
E (eV)	I (arb. units)	E (eV)	I (arb. units)
2465.857	0.0046	2465.857	0.0046
2465.857	0.0046	2465.857	0.0046
2469.824	0.0011	2469.813	0.0011
2470.294	0.0000	2470.266	0.0000
2471.087	0.0007	2471.050	0.0006
2471.087	0.0007	2471.050	0.0006

Table S17: CVS-CCSD/un-aug-pc-3 stick spectrum for H_2S. Relativistic shift is not included.

E (eV)	I (arb. units)
2468.753	0.0063
2469.622	0.0032
2472.394	0.0015
2472.512	0.0006
2472.534	0.0003
2472.622	0.0019

Table S18: CVS-CCSD/un-aug-pc-3 stick spectrum for SO_2. Relativistic shift is not included.

E (eV)	I (arb. units)
2469.195	0.0094
2474.195	0.0046
2475.214	0.0056
2476.944	0.0003
2477.789	0.0010

Table S19: CVS-CCSD/un-aug-pc-3 stick spectrum for OCS. Relativistic shift is not included.

E (eV)	I (arb. units)
2469.460	0.0034
2469.460	0.0034
2469.918	0.0056
2471.867	0.0002
2473.268	0.0012
2473.280	0.0011
2473.280	0.0011

Table S20: CVS-CCSD/un-aug-pc-3 stick spectrum for CS. Relativistic shift is not included.

E (eV)	I (arb. units)
2467.650	0.0051
2467.650	0.0051
2471.719	0.0017
2472.654	0.0001
2473.718	0.0011
2473.718	0.0011

Table S21: CVS-CC3/un-aug-pc-3 stick spectrum for H_2S. Relativistic shift is not included.

E (eV)	I (arb. units)
2466.481	0.0021
2468.796	0.0003
2468.881	0.0001
2469.054	0.0011
2470.196	0.0005

Table S22: CVS-CC3/un-aug-pc-3 stick spectrum for CS. Relativistic shift is not included.

E (eV)	I (arb. units)
2464.148	0.0040
2464.148	0.0040
2468.777	0.0010
2469.320	0.0000
2470.038	0.0006
2470.038	0.0006

Table S23: CVS-CC3/un-aug-pc-3 stick spectrum for OCS. Relativistic shift is not included.

E (eV)	I (arb. units)
2467.075	0.0035
2468.293	0.0009
2469.363	0.0006
2469.363	0.0006
2469.392	0.0006
2470.390	0.0003
2470.390	0.0003
